# SOD1 Promotes Cell Proliferation and Metastasis in Non-small Cell Lung Cancer via an miR-409-3p/SOD1/SETDB1 Epigenetic Regulatory Feedforward Loop

**DOI:** 10.3389/fcell.2020.00213

**Published:** 2020-04-23

**Authors:** Shilong Liu, Bin Li, Jianyu Xu, Songliu Hu, Ning Zhan, Hong Wang, Chunzi Gao, Jian Li, Xiangying Xu

**Affiliations:** ^1^Department of Thoracic Radiation Oncology, The Third Affiliated Hospital of Harbin Medical University, Harbin, China; ^2^Department of Plastic Surgery, Nanfang Hospital of Southern Medical University, Guangzhou, China; ^3^Department of Radiation Oncology, Xiang’an Hospital of Xiamen University, Xiamen, China; ^4^College of Bioinformatics Science and Technology, Harbin Medical University, Harbin, China; ^5^Department of Oncology, The First Affiliated Hospital of Harbin Medical University, Harbin, China; ^6^Department of Radiation Oncology, The Third Affiliated Hospital of Sun Yat-sen University, Guangzhou, China

**Keywords:** non-small cell lung cancer, SOD1, miR-409-3p, SETDB1, methylation, feedforward loop

## Abstract

Superoxide dismutase 1(SOD1) is a major antioxidant with oncogenic effects in many human cancers. Although SOD1 is overexpressed in various cancers, the clinical significance and functions of SOD1 in non-small cell lung cancer (NSCLC), particularly the epigenetic regulation of SOD1 in NSCLC carcinogenesis and progression have been less well investigated. In this study, we found that SOD1 expression was upregulated in NSCLC cell lines and tissues. Further, elevated SOD1 expression could promote NSCLC cell proliferation, invasion and migration. While inhibition of SOD1 expression induced NSCLC G1-phase cell cycle arrest and promoted apoptosis. In addition, miR-409-3p could repress SOD1 expression and significantly counteract its oncogenic activities. Bioinformatics analysis indicated that SET domain bifurcated histone lysine methyltransferase1 (SETDB1) was involved in the epigenetic regulation of miR-409-3p and SOD1 expression and functions in NSCLC cells. Identification of this miR-409-3p/SOD1/SETDB1 epigenetic regulatory feedforward loop may provide new insights into further understanding of NSCLC tumorigenesis and progression. Additionally, our results incicate that SOD1 may be a potential new therapeutic target for NSCLC treatment.

## Introduction

Due to advances in clinical and experimental oncology, particularly the successful reduction of smoking prevalence, the incidence rate of lung cancer has continued to decline in recent decades, Nevertheless, the prognosis for patients with non-small cell lung cancer (NSCLC) remains unfavorable. Lung cancer remains among the leading causes of cancer death with a 5-year survival rate of only 18% across all ethnicities and disease stages (Siegel et al., 2018). Lung cancer, particularly NSCLC, has usually reached an advanced stage when first diagnosed. Hence, early diagnosis and treatment are vital for improving survival rates, and substantial efforts have been made to identify molecular markers that can predict patient prognosis (Zhou et al., 2016), however, additional new treatment strategies are still needed.

Superoxide dismutases (SODs) are enzymes required for the conversion of superoxide into oxygen and hydrogen peroxide. There are three SOD subtypes: SOD1, SOD2, and SOD3. Among these three SOD enzymes, SOD1 (Cu/Zn superoxide dismutase) is the major cytosolic form and provides 80% of total cellular SOD activity (Watanabe et al., 2014). Although SOD1 is primarily distributed in the cytoplasm, it is also found in the mitochondrial intermembrane space and the nucleus (Tsang et al., 2014). The function of SOD2 and SOD3 are similar to that of SOD1, but they are located in different cell compartments. In response to oxidative stress, SOD1 maintains low levels of superoxide to protected the cell from death in response to oxidative stress damage (Tsang et al., 2014). The antioxidant effect of SOD1 can also provide protection for cancer cells or other dysfunctional cells. Mutations of the *SOD1* gene have been linked to numerous human diseases and cancers, such as and Down syndrome and familial amyotrophic lateral sclerosis (ALS), Indeed 20% of ALS cases are associated with mutations in SOD1 (Brasil et al., 2019), Somwar et al. (2011) reported that SOD1 was overexpressed in lung adenocarcinomas when compared with the normal lung tissue, while Glasauer et al. (2014) found that inhibition of SOD1 by the small molecule ATN-224 induced NSCLC cell death.

SOD1 also acts as a metabolic focal point, integrating O_2_, nutrients, and reactive oxygen species (ROS) to direct energy metabolism (Tsang et al., 2018). Deficiency of SOD1 decreased the lifespan and accelerated aging in SOD1(−/−) mouse model (Watanabe et al., 2014; Zhang et al., 2017). Furthermore, the SOD1 inhibitor, ATN-224, has been tested in phase 1 clinical trials in patients with solid tumors (Lowndes et al., 2008) and in phase 2 clinical trials for prostate cancer (Lin et al., 2013), however, there have been few reports on the clinical significance of SOD1 functions in lung cancer, particularly the mechanism underlying the role of SOD1 in progression and carcinogenesis.

MicroRNAs make up a class of small non-coding RNAs that regulate gene expression at the post-transcriptional level through binding to specific sequences through binding to specific in the 3′untranslated regions (3′UTRs) of target mRNAs, leading to transcript degradation or translational inhibition (Lu and Clark, 2012). Dysregulation of miRNAs is involved in numerous human biological and pathological processes, including cell proliferation, differentiation, development, apoptosis, and tumorigenesis (Wu et al., 2019). miR-409-3p, maps to chromosome 14q32.31, and has been shown significantly downregulated in lung adenocarcinoma tissues when compared with corresponding noncancerous tissues, and can inhibit growth, migration, and invasion, as well as inducing apoptosis in lung adenocarcinoma cells via inactivation of Akt signaling by targeting c-Met (Wan et al., 2014).

In our study, we found that SOD1 expression levels are significantly increased in NSCLC compared with normal lung tissues and cells using bioinformatic and laboratory experiments. Furthermore, high levels of SOD1 promoted lung cancer cell proliferation and metastasis, while miR-409-3p inhibited SOD1 activity through binding to its 3′ UTR. We also found that SET domain bifurcated histone lysine methyltransferase 1 (SETDB1) may contribute to the interaction between miR-409-3p and SOD1 by an epigenetic transcription factor.

## Materials and Methods

### Clinical Tissue Samples and Cell Lines

Tissue specimens (*n* = 196) obtained from patients diagnosed with stage I–IIIb NSCLC who underwent surgery at The Third Affiliated Hospital of Harbin Medical University between March 2007 and December 2009 were used for immunohistochemical staining. Eighteen pairs of NSCLC tumor and adjacent normal tissue samples were collected during surgery between April and August 2016, immediately frozen in liquid nitrogen and stored at −80°C for further analysis. None of the patients underwent any therapy before surgery. Informed consent was obtained from all patients. The study was approved by the Ethics Committee of The Third Affiliated Hospital of Harbin Medical University.

Seven NSCLC cell lines [A549, PC-9, NCI-H1299, NCI-H460, NCI-H1650, NCI-H520 and human bronchial epithelial cells (16HBE)] were purchased from American Type Culture Collection (ATCC, Manassas, VA, United States). PC-9 and 16HBE cells were cultured in DMEM (GIBCO, Invitrogen, Carlsbad, CA, United States), other cells were cultured in RPMI 1640 basic medium (GIBCO, Invitrogen, Carlsbad, CA, United States), supplemented with 10% fetal bovine serum (FBS; Invitrogen, Carlsbad, CA, United States) and antibiotics (Invitrogen, Carlsbad, CA, United States) at 37°C in a 5% CO_2_ atmosphere.

### Immunohistochemistry

Tissue sections were deparaffnized in xylene and rehydrated in a series of graded alcohol solutions according to standard procedures. Antigen retrieval was performed by placing the slides in citrate buffer (0.01 M, pH 6.0) heated for 10 min and cooled down naturally to enhance immunoreactivity. The slides were immersed in hydrogen peroxide (3%) for 15 min at room temperature. the specimens were then incubated with the SOD1 primary antibody (1:100, rabbit polyclonal; wanleibio china) at 4°C overnight. After incubation with biotin-labeled secondary antibodies goat anti-rabbit IgG antibodies (1:200 Beyotime A0277 china), at room temperature for 30 min, the specimens were incubated in Horseradish Peroxidase-labeled Avidinat 37°C for 30 min. The slides were stained using DAB and counterstained using hematoxylin. Finally, each section was dehydrated by gradient alcohol and covered with a coverslip. The staining results were analyzed by two independent pathologists experienced in evaluating IHC, both of whom were blinded to the clinicopathological data. The staining results were scored according to the following criteria: the sum of intensity and proportion of positively stained cells. The staining intensity was classifed: 0 (no staining), 1 (light yellow/weak staining), 2 (yellow-brown/moderate staining), or 3 (brown/strong staining). The proportion of positively stained tumor cells was scored as follows: 1 (positive cells 1–25%), 2 (positive cells 26–50%), 3 (positive cells 51–75%), or 4 (positive cells 75–100%). Thus, the final score ranged from 0 to 7, and the median value of score 3 was used to distinguish low versus high SOD1 expression. The specimen with a final score of 3 was classifed as having low expression. Otherwise, the specimen was classifed as having high expression. Any discrepancies between scores were reviewed by the two pathologists plus a senior pathologist until a consensus was reached.

### RNA Extraction and Quantitative Reverse Transcription Polymerase Chain Reaction (qRT-PCR) Assays

Total RNA was extracted from tissue samples and cells using Trizol Reagent (Invitrogen, Carlsbad, CA, United States), cDNA was synthesized using the Transcriptor First Strand cDNA Synthesis Kit (Roche, Mannheim, Germany), and qRT-PCR performed using a Fast Start Universal SYBR Green Master (ROX) (Roche, Mannheim, Germany) on an ABI7500 system, according to the manufacturer’s instructions. Results were normalized to GAPDH expression levels. Fold-change in gene expression levels were calculated using the 2^–ΔΔCt^ method.

### Cell Transfection

Cells were transfected using Lipofectamine 2000 (Invitrogen, Carlsbad, CA, United States), following the manufacturer’s instructions. Three *SOD1*-specific si-RNA oligonucleotides (si-SOD1-1: 5′-TTC GAG CAG AAG GAA AGT AAT GGA CCA-3′,si-SOD1-2: 5′-GCA GAG GGA GAA TGC TTA GCA-3′, and si-SOD1-3: 5′-GGCCTGCATGGATTCCATG-3′) and scrambled negative control (FAM-F: 5′-GUC ACA CGG GAA GAG AGU UAA AGA CUA -3′ R: 5′-GGA UAU GGG AAG AGC GUA GUU AAU-3′) were designed and purchased from Invitrogen (Carlsbad, CA, United States). The *SOD1*sequence, 5′-TCCCTTGGATGTAGTCTGAGGACTCCATT-3′ was synthesized and cloned into the pLVX-puro vector. Lentiviral particles were constructed and packaged by GeneChem Co., Ltd. (Shanghai, China). miR-409-3p mimic (5′-GAAUGUUGCUCGGUGAACCCCU-3′) and a negative control (5′-ACTACTGAGTGACAGTAGA-3′) were also obtained from GeneChem Co., Ltd. (Shanghai, China). Sequences of other primers are provided in Table 1.

**TABLE 1:** Oligonucleotides used in this study.

Gene	Orientation	5′- 3′ primer sequence
miR-409-3p inhibitor	Forward	GAATGTTGCTCGGTGA
	Reverse	GTGCAGGGTCCGAGGT
*U6*	Forward	GCTTCGGCAGCACATATACT
	Reverse	GTGCAGGGTCCGAGGTATTC
*GAPDH*	Forward	GCACCGTCAAGGCTGAGAC
	Reverse	TGGTGAAGACGCCAGTGGA
*SOD1*	Forward	CTGAAGGCCTGCATGGATTC
	Reverse	CCAAGTCTCCAACATGCCTCT

### Cell Proliferation Assay

Cell viability was assessed using a Cell Counting Kit-8 (CCK-8; Dojindo, Kumamoto, Japan), according to the manufacturer’s protocol. Briefly, cells were plated in 96-well plates at a density of 2 × 10^3^ cells per well, and incubated for 24, 48, 72, or 96 h, and treated with 10 μl/well of CCK-8 solution at the indicated time points after 2 h of incubation, and cell proliferation curves were plotted based on absorbance values (OD = 450 nm) at each time point. The assay was conducted using five replicate wells per sample and three parallel experiments were performed.

### Flow Cytometry Assay

An AnnexinV-PE Apoptosis Detection Kit (BD Biosciences, San Jose, CA, United States) was used to examine apoptosis, according to the manufacturer’s instructions. Briefly, cells were washed twice in cold phosphate-buffered saline (PBS) and then harvested and resuspended in 1 × binding buffer. Next, 100 μl of suspended cells (1 × 10^5^ cells) was transferred to a 5 ml culture tube, and 5 μl of annexin V-PE and 5 μl of (propidium iodide) PI were added. Cells were gently vortexed and incubated for 15 min at room temperature in the dark, followed by addition of 400 μl 1 × binding buffer to each tube. For cell cycle analysis, the CycleTEST^TM^ PLUS DNA Reagent Kit (BD Biosciences. Cat No. 340242) was used. Briefly, cells were washed with ice-cold PBS and fixed with 75% ethanol overnight at −20°C. After fixation, cells were washed and resuspended twice in PBS and then incubated with PI and RNase for 30 min at room temperature. A Canto II flow cytometer (BD Biosciences, San Jose, CA, United States) was used to evaluate levels of apoptosis and the cell cycle in each sample, following the manufacturer’s instructions.

### Cell Invasion and Migration Assay

Cells were plated in serum-free medium in the upper chamber of transwell plates (8-μm pore size, Corning, Tewksbury, MA, United States). For the invasion assay, transwell membranes were precoated with 45 μg matrigel (Sigma Aldrich, United States) to form a matrix barrier, while matrigel was not used for migration assay. Standard medium supplemented with 15% FBS was added to the bottom chambers. After incubation at 37°C for 12 h (migration) or 24 h (invasion), cells that had migrated to the lower membranes surfaces were fixed in 90% ethanol for 30 min, stained with 0.5% crystal violet, and photographed under microscope. Cell numbers in five random fields in each well were counted under a light microscope (magnification, × 200) and the number of invasion or migration cell normalized to the total cell number, normalized invasion cell or migration cell number = actual invasion cell or migration cell number/each cell growth rate. Each experiment was performed in triplicate.

### Luciferase Reporter Assay

A *SOD1* 3′-UTR sequence containing an miR-409-3p binding site was cloned and inserted into the pmiR-RB-luciferase reporter vector (Promega, United States), to generate the *SOD1* 3′-UTR-WT construct. Mutation of the *SOD1* miR-409-3p binding site was conducted by site-directed mutagenesis using the Quick Change Lightning kit (Stratagene, La Jolla, CA, United States) to generate the SOD1 3′-UTR-MUT construct. For the experiments: cells were plated in 24-well plates, incubated overnight, and then H460 cells co-transfected with SOD1 3′-UTR-WT, SOD1 3′-UTR-MUT, miR-409-3p mimic, or miR-409-3p inhibitor. Luciferase activity was tested using the Dual-Luciferase Reporter Assay System (Promega, United States). Renilla activity was used to normalized luciferase activity values for each sample.

### Western Blot Analysis

Protein samples were extracted using RIPA lysis buffer containing 1% phenylmethanesulfonyl fluoride. Protein concentrations in lysates were measured using the protein BCA assay kit (Waileibio WAL004,China) and aliquots containing 40 μg protein separated by 10% sodium dodecyl sulfate polyacrylamide gel electrophoresis (SDS-PAGE), and transferred to polyvinylidene fluoride membranes. Then, membranes were incubated with SOD1 antibody (1:500) overnight at 4°C, followed by incubation with horseradish peroxidase-labeled goat anti-rabbit IgG antibody (1:1000). Bound antibodies were detected using an ECL Western Blotting Detection system and SOD1 levels quantified by densitometric analysis of the protein bands relative to total protein loaded, using Gel-Pro-Analyzer software. β-actin was used as the loading control. All antibodies were purchased from Wanlei Bio (Shenyang, China).

### Statistical Analysis

All data are presented as mean ± standard deviation (SD) from three independent experiments. Statistical analyses were performed using GraphPad Prism version 6.0 (GraphPad Software Inc., La Jolla, CA, United States), R software, or SPSS 21.0 for Windows (SPSS, Chicago, IL, United States). Clinicopathological data were analyzed using the chi-square test and survival curves were assessed by Kaplan-Meier analysis. The Cox proportional hazards model was used to identify independent prognostic factors for overall survival (OS) and disease-ress survival (DFS). Quantitative variables were analyzed using the Student’s *t*-test, while the chi-square or Fisher’s exact tests were used to compare qualitative variables. *P*-values < 0.05 were considered statistically significant.

## Results

### Expression of SOD1 Is Elevated in NSCLC Tissues and Cell Lines

**FIGURE 1:**
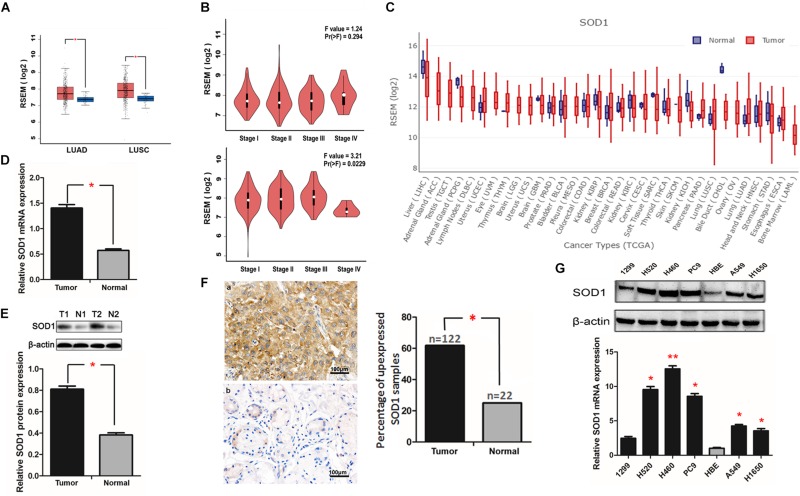
Superoxide dismutase 1 (SOD1) expression is elevated in non-small cell lung cancer (NSCLC) tissues and cell lines. **(A,B)** SOD1 was overexpressed in both ADC and SCC tumors relative to normal tissues, and its expression also differed according to TNM stage as recorded in TCGA database. **(C)** Pan-cancer analysis of SOD1 expression. **(D,E)** Representative qRT-PCR and western blot analysis of SOD1 expression in 18 paired NSCLC clinical tissue specimens. Histogram of pooled data from NSCLC (T, *n* = 18) and adjacent normal lung (N, *n* = 18) tissue samples. Data are expressed as means ± standard deviation (SD). ^∗^*P* < 0.05. **(F)** Representative immunohistochemistry images of NSCLC (T) and adjacent normal lung (N) tissue samples. Significantly darker brown staining of SOD1 protein was detected in cancer tissues **(a)** than in adjacent normal tissues **(b)**. Histogram of pooled data from NSCLC (tumor, *n* = 122) and normal lung (normal, *n* = 22) tissue. The percentage of NSCLC tissues with high SOD1 expression was significantly greater than that of normal lung tissues; ^∗^*P <* 0.05. **(G)** qRT-PCR and western blot analysis of SOD1 expression in NSCLC cell lines and a normal bronchial epithelial cell line. The histogramillustrates of overexpression of SOD1 mRNA in NSCLC tumor tissues relative to normal lung tissues; ^∗^*P* < 0.05, ^∗∗^*P* < 0.01.

First, we used an algorithm to extract expression data for *SOD1* in NSCLC from The Cancer Genome Atlas (TCGA) database. *SOD1* expression was significantly higher in lung adenocarcinoma (ADC) and lung squamous cell carcinoma (SCC) than in normal tissues (Figure 1A). Further data analysis demonstrated that *SOD1* expression also differed according to TNM stage (Figure 1B). In addition, we found that mass pan-cancer *SOD1* expression levels differed from those in normal tissues (Figure 1C), also the *SOD1* expression levels varied in different normal tissues (Supplementary Figure S1).

Next, we used qRT-PCR, western blotting, and immunohistochemical staining to verify our findings regarding SOD1 expression in NSCLC tissue samples. Eighteen pairs of cancer and corresponding adjacent normal tissue samples were used for qRT-PCR and western blot assays. The results showed that both mRNA and protein expression levels of SOD1 expression in cancer tissues were significantly higher than those in adjacent normal tissues (Figures 1D,E). Furthermore, immunohistochemical staining results show a significantly darker brown staining SOD1 in cancerous than adjacent tissues; with SOD1 expression was upregulated in 62.2% (122/196) of tumor tissues but only in 12.2% (22/196) of normal tissue samples (*p* < 0.05). These results confirm that SOD1 expression is significantly increased in tumors relative to adjacent normal tissues (Figure 1F and Supplementary Figure S2).

Next, we used qRT-PCR and western blotting to verify the differential expression of SOD1 in human NSCLC cell lines and a normal human bronchial epithelial cell line. The results confirmed that SOD1 was expressed at significantly higher levels in NSCLC than normal bronchial epithelial cells at both the mRNA and protein levels (Figure 1G and Supplementary Figure S3).

### Association of SOD1 With Clinicopathological Characteristics in Patients With NSCLC

Immunohistochemical analysis confirmed that high expression of SOD1 was associated with larger tumor size (*p* = 0.011), lymph node metastasis (*p* < 0.001), and advanced pTNM stage (*p* < 0.001) in NSCLC. SOD1 was highly expressed in 62.2% (122/196) of NSCLC tissue samples, including 35.2% of ADC and 64.8% of SCC specimens (Table 2). No significant correlation was observed between SOD1 expression and age, sex, differentiation or smoking status.

**TABLE 2:** Association between SOD1 expression and clinicopathological characteristics patients with NSCLC.

		SOD1 expression		
Variable	All patients	High (%)	Low (%)	*P*
			
	(*n* = 196)	(*n* = 122)	(*n* = 74)	
**Smoking**				
Never	80	53 (43)	27 (36)	0.337
Ever	116	69 (57)	47 (64)	
**Gender**				
Male	129	79 (65)	50 (68)	0.687
Female	67	43 (35)	24 (32)	
**Age (years)**				
<60	89	54 (44)	35 (47)	0.679
≥60	107	68 (56)	39 (53)	
**Differentiation**				
Well	20	11(9)	9 (12)	0.846
Moderate	75	46 (37)	29 (39)	
Poor	101	65 (44)	36 (49)	
**Histological cell type**				
Adenocarcinoma	74	43 (35)	31 (42)	0.352
Squamous cell carcinoma	122	79 (65)	43 (58)	
**PStage**				
I	93	45 (37)	48 (65)	< 0.001^∗^
II	40	25 (20)	15 (20)	
III	63	52 (43)	11 (15)	
**pT classification**				
T1	34	14 (11)	20 (27)	0.011^∗^
T2	129	82 (67)	47 (64)	
T3/4	33	27 (22)	7 (9)	
**Lymph node metastasis**				< 0.001^∗^
N0	114	56 (46)	58 (78)	
N1	26	17 (14)	9 (12)	
N2	56	49 (40)	7 (10)	
**Adjuvant chemotherapy**				
Yes	121	76 (62)	45 (61)	0.836
No	75	46 (38)	29 (39)	
**Adjuvant radiotherapy**				
Yes	89	59 (48)	30 (41)	0.286
No	107	63 (52)	44 (59)	

*Abbreviations: NSCLC, non-small cell lung cancer; pTNM stage, tumor node metastasis (pathological stage); pT, pathological T stage; n, number of patients. Ever, smoking at any time from the beginning of life. P-value, the difference of clinicopathological characteristics between the SOD1 high expression group and low expression group. ^∗^P < 0.05 was considered statistically significant.*

### SOD1 Expression Can Predict Survival Prognosis in Patients With NSCLC

Next, we used univariate and multivariate COX regression analysis to determine whether SOD1 expression is related to survival in patients with NSCLC (Table 3). Univariate analysis indicated that advanced TNM stage, larger tumor size, lymph node metastasis, no adjuvant chemotherapy, and high SOD1 expression levels were factors associated with inferior overall survival (OS) with multivariate analysis, TNM stage (HR, 2.421; 95% CI, 1.221–4.801; *P* = 0.001), and SOD1 expression level (HR, 1.858; 95% CI, 1.164–2.966; *P* = 0.009) were identified as independent prognostic factors for OS. In addition, univariate analysis of disease-free survival (DFS) of patients with NSCLC showed that advanced TNM stage, large tumor, lymph node metastasis, no adjuvant chemotherapy, no adjuvant radiotherapy, and high SOD1 expression predicted poorer DFS, while TNM stage (HR, 2.055; 95% CI, 1.100–3.840; *p* = 0.024), adjuvant radiation therapy (HR, 0.497; 95% CI, 0.263–0.940; *P* = 0.032), and SOD1 expression (HR, 1.605; 95% CI, 1.056–2.440; *P* = 0.027) were independent prognostic factors for DFS on multivariate analysis.

**TABLE 3:** Univariate and multivariate analyses of overall survival and disease-free survival.

	OS			DFS		
	Univariate analysis	Multivariate analysis		Univariate analysis	Multivariate analysis	
Variable	*P*	HR (95% CI)	*P*	*P*	HR (95% CI)	*P*
**Age**						
<60						
≥60	0.245	−	−	0.453	−	−
**Gender**						
Female						
Male	0.106	−	−	0.368	−	−
**Smoking**	0.126	−	−	0.103		
Never						
Ever						
**Differentiation**						
Good						
Moderate						
Poor	0.309	−	−	0.181	−	−
**Histological cell type**						
*Squamous cell carcinoma*						
Adenocarcinoma	0.249	−	−	0.062	−	−
**pT stage**						
I						
II						
III	0.152	−	−	0.002^∗^	1.334 (0.958 to 1.859)	0.088
**pTNM stage**						
I						
II						
III	< 0.001^∗^	2.421 (1.221 to 4.801)	0.011^∗^	< 0.001^∗^	2.055 (1.100 to 3.840)	0.024^∗^
**Lymph node metastasis**						
Present						
Absent	< 0.001^∗^	0.928 (0.561 to 1.534)	0.771	0.001^∗^	0.881(0.547 to1.419)	0.602
**Adjuvant chemotherapy**						
No						
Yes	0.003^∗^	0.982 (0.496 to 1.946)	0.959	0.018^∗^	1.154(0.657 to2.028)	0.618
**Adjuvant radiotherapy**						
No						
Yes	0.165	−	−	0.008^∗^	0.497(0.263 to 0.940)	0.032^∗^
**SOD1 expression**						
Low	< 0.001^∗^	1.858 (1.164 to 2.966)	0.009^∗^	0.002^∗^	1.605 (1.056 to 2.440)	0.027^∗^
High						

*Abbreviations: NSCLC, non-small cell lung cancer; pTNM stage, tumor node metastasis (pathological stage); pT, pathological T stage; n, number of patients; OS, overall survival; DFS, disease-free survival; HR, hazard ratio; CI, confidence interval. pTNM stage, lymph node metastasis, and SOD1 expression were selected for inclusion in multivariate analysisfor OS. Histological cell type, pTNM stage, lymph node metastasis, and SOD1 expression were selected for inclusion in multivariate analysis for DFS. ^∗^P < 0.05 was considered statistically significant.*

**FIGURE 2:**
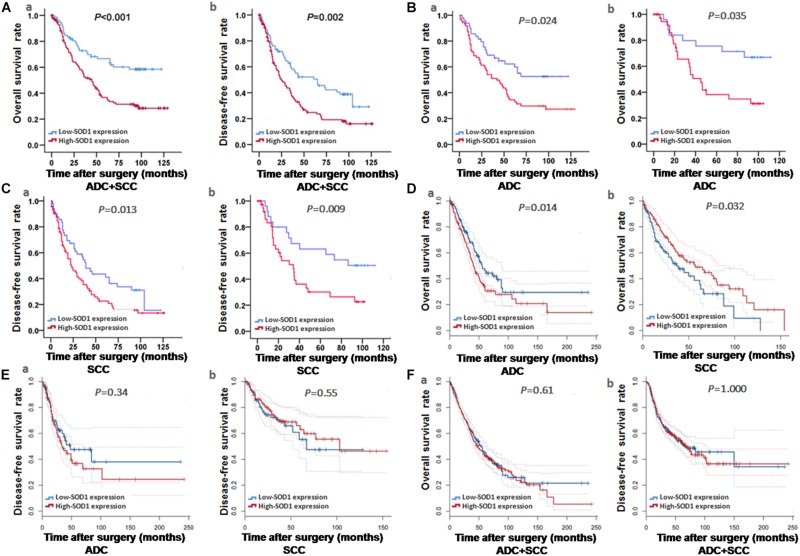
Kaplan-Meier curve analysis of survival rates in patients with adenocarcinoma (ADC) and squamous cell carcinoma (SCC), based on experimental and bioinformatics data. **(A–C)** Kaplan-Meier curve analysis of Overall survival (OS) and disease-free survival (DFS) based on our exprimental data. OS and DFS of patients with ADC or SCC exhibiting high SOD1 protein expression were significantly poorer to those exhibiting low SOD1 protein levels. These results were consistent with subgroup analysis of OS and DFS in ADC and SCC. **(D–F)** Kaplan-Meier curve analysis of OS and DFS based on bioinformatics data. OS was significantly reduced in patients with high than in those with low SOD1 protein expression in ADC (*P* = 0.014 **D-a**), but the OS in SCC patients was positively correlated with SOD1 expression (*P* = 0.032, **D-b**); In addition, no significant differences between high and low SOD1 expression levels in ADC **(E-a)** and SCC **(E-b)**. **(F)**. No significant differences were also observed in total OS **(F-a)** and DFS **(F-b)** for NSCLC (including ADC and SCC). *P* < 0.05 was considered significant.

Kaplan-Meier analysis revealed that high levels of SOD1 expression was correlated with poor prognosis of both OS (χ2 = 20.37; *P* < 0.001, Figure 2A-a) and DFS (χ2 = 20.37; *p* < 0.001, Figure 2A-b) in our patient population. Further stratified analysis showed that SOD1 levels were significantly negatively correlated with OS (χ2 = 6.827; *P* = 0.009, Figure 2B-a) and DFS (χ2 = 4.467; *P* = 0.035, Figure 2C-a) in ADC as well as with OS (χ2 = 6.182; *P* = 0.013, Figure 2B-b) and DFS (χ2 = 5.062; *P* = 0.024, Figure 2C-b) in SCC.

To further verify the impact of SOD1 levels on prognosis in patients with NSCLC, we used the GEPIA analysis tool (Tang et al., 2017) to evaluate the expression levels of SOD1 and NSCLC survival progression in large database, and we found that SOD1 expression was significantly negatively correlated with OS in patients with ADC (*P* = 0.014, Figure 2D-a), but positively correlated with SOD1 expression the OS in SCC patients (*P* = 0.032, Figure 2D-b). This result is likely to be related to the significantly reduced expression of SOD1 in stage IV SCC. Nevertheless, No significant association was found between SOD1 levels and DFS in patients with ADC or SCC (*P* = 0.34, Figure 2E-a and *P* = 0.55, Figure 2E-b, respectively), or between SOD1 levels and OS and DFS in patients with NSCLC overall using this dataset (*P* = 0.86, Figure 2F-a and *P* = 0.65, Figure 2F-b, respectively).

### SOD1 Can Promote the Proliferation, Invasion, and Migration of NSCLC Cells

**FIGURE 3:**
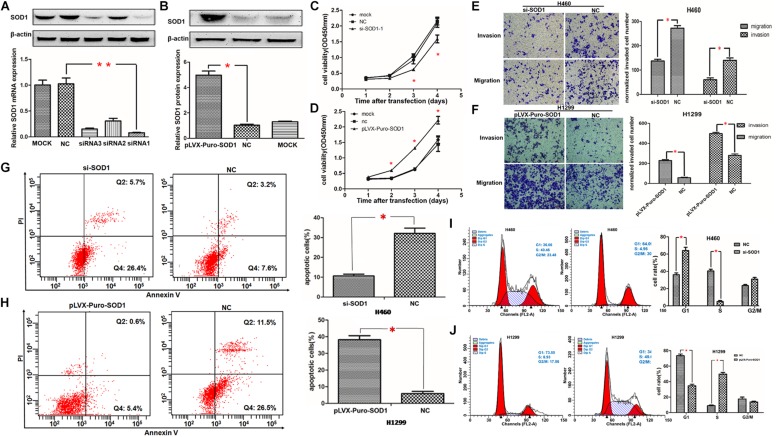
Regulation of NSCLC cell proliferation, invasion, metastasis, apoptosis, and the cell cycle by SOD1. **(A,B)** SOD1 mRNA and protein expression levels were analyzed following transfection of SOD1 siRNA or pLVX-puro-SOD1 into H460 and H1299 cells. Representative western blot results are shown. **(C,D)** Proliferation of H460 and H1299 cells analyzed by CCK-8 assay after inhibition or overexpression of SOD1. **(E,F)** Evaluation of H460 and H1299 cell migration and invasion by transwell assay after inhibited or overexpressed of SOD1. **(G–J)** Measurement of apoptosis and the cell cycle by flow cytometryin cells with upregulated or downregulated SOD1 expression. Experiments were conducted using triplicate samples and each experiment was conducted three times; ^∗^*P* < 0.05, ^∗∗^*P* < 0.01.

Based on our findings of elevated SOD1 expression in NSCLC cell lines, we selected the highest (H460) and lowest (H1299) expressing cell lines for transfection with si-SOD1 and pLVX-puro-SOD1, respectively. The knockdown efficiency of si-SOD1-1 in H460 cells was most significant test at both the mRNA and protein levels (Figure 3A and Supplementary Figure S4), while SOD1 expression was significantly upregulated in H1299 cells after pLVX-puro-SOD1 transfection (Figure 3B); subsequently, we used these two cell lines to perform loss- and gain-of-function experiments, respectively.

Proliferative capacity is essential for the growth and development of malignant tumors; therefore, we used the CCK-8 assay to test the effect of SOD1 on NSCLC cell proliferation. Compared with controls and normals, knockdown of SOD1 significantly reduced proliferation ability of H460 cells (Figure 3C). In contrast, cell proliferation was significantly enhanced in H1299 cells over-expressing SOD1 (Figure 3D).

Migration and invasion are vital for tumor progression and metastasis. Here, we used transwell assays to assess cell invasive and migratory ability. According the cell proliferation rate can affect the cell number went through the trans-well, we use the respective cell proliferation rates to normalized the number of migrate and invaded cells, and use this to evaluate the ability of migration and invasion. Knockdown of SOD1 significantly repressed the invasion and migration of H460 cells, compared with controls and normals (Figure 3E). Conversely, overexpression of SOD1 markedly enhanced the invasive and migratory of H1299 cells (Figure 3F).

### SOD1 Reduces Apoptosis and Promotes Cell Cycle Progression of NSCLC Cells

To further explore the mechanism underlying how SOD1 enhances NSCLC cell proliferation, we examined the effect of SOD1 on apoptosis and the cell cycle of NSCLC cells using flow cytometry. Knockdown of SOD1 expression led to significantly increases of both early and late apoptotic H460 cells, relative to untreated control cells (Figure 3G). In contrast, overexpression of SOD1 in H1299 cells led to a significantly decreased apoptosis rate (Figure 3H). Regarding the cell cycle, flow cytometry revealed that inhibition of SOD1 result in G1-phase arrest, We observed a significant increase in the number of cells in G1-phase and a concomitant significant decrease in those in S-phases, relative to control group H460 cells (Figure 3I). Conversely, overexpression of SOD1 in H1299 cells promoted cell cycle progression, reducing the proportion of cells in G1-phase and increasing the proportion of those in S phases (Figure 3J).

### SOD1 Expression Can Be Regulated by miR-409-3p

**FIGURE 4:**
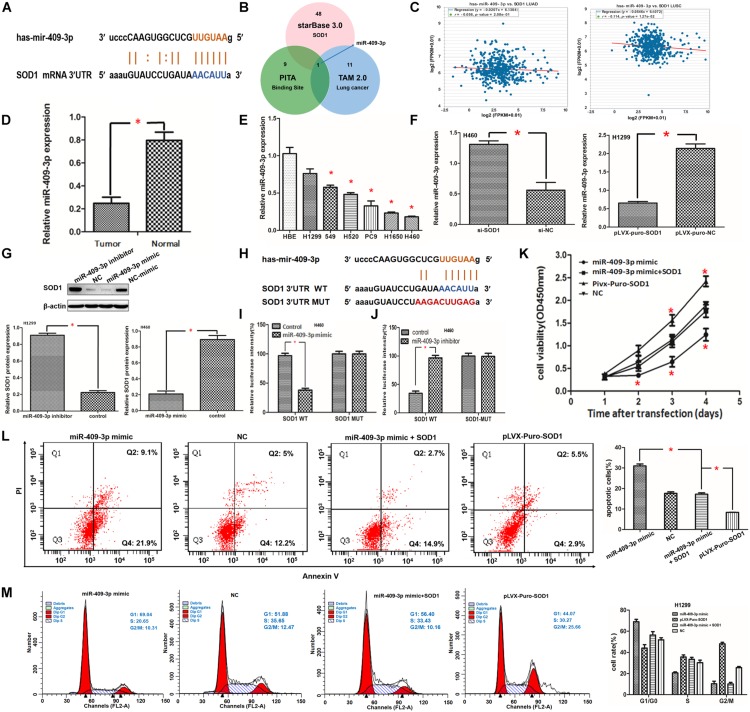
**(A,B)** Superoxide dismutase 1 (*SOD1)* mRNA may be a target of miR-409-3p. Binding sites for miR-409-3p in the SOD1 promoter predicted using the bioinformatics website (starBasev3.0 including TAM and PITA) are shown. **(C)** Relationship between the expression of miR-409-3p and *SOD1* level in starBasev3.0. **(D,E)** qRT-PCR showing that miR-409-3p expression was inhibited in NSCLC tissue and cells. **(F)** Relative expression profiles of miR-409-3p in NSCLC cells with or without SOD1 inhibition. **(G)** Representative western blot assay and histogram demonstrating relative SOD1 expression in NSCLC cells transfected with miR-409-3p inhibitor or miR-409-3p mimic. **(H)** Wild-type (WT) or mutant miR-409-3p binding sequences in the SOD1 promoter were constructed using starBase v3.0. **(I,J)** Dual-luciferase reporter gene assay to determine luciferase activity from wild-type (WT) or mutant SOD1 promoter sequences 48 h after transfection. **(K,L)**The effects of SOD1 on proliferation and apoptosis of NSCLC cells was rescued by overexpression of miR-409-3p; however, the effect of SOD1 on cell cycle phase distribution was not significantly affected by miR-409-3p mimic **(M)**. Each experiment was conducted in triplicate; ^∗^*P* < 0.05.

MicroRNAs are critical for tumor occurrence and progression and can act as oncogenes or tumor suppressor genes. Here, we identified 49 microRNAs related to SOD1 in starBase v3.0 (Li et al., 2014), and used the TAM database to functionally annotate them. Twelve microRNAs related to NSCLC were identified. Finally, only miR-409-3p was predicted to bind to the *SOD1* 3′ UTR (Figures 4A,B). Furthermore, starBase v3.0 data analysis indicated that expression of *SOD1* and miR-409-3p was negatively correlated in NSCLC, particularly in SCC (*P* = 0.0127, Figure 4C). The data indicated that miR-409-3p might inhibit *SOD1* expression. So we speculate that *SOD1* may be a target mRNA of miR-409-3p.

### miR-409-3p Inhibits SOD1 Expression Through Direct Interaction

To verify the bioinformatics data indicating that *SOD1* is a target of miR-409-3p, we designed additional experiments. First, we evaluated 18 pairs of cancerous and corresponding normal adjacent tissue samples from patients with NSCLC. Total RNA was extracted for qRT-PCR analysis and the results showed that miR-409-3p was significantly decreased in NSCLC relative to adjacent normal tissues (Figure 4D). In addition, we compared the expression of miR-409-3p in NSCLC and normal human bronchial epithelial cell lines. The results confirmed that miR-409-3p is expressed at significantly lower levels in NSCLC cells (Figure 4E). These findings are consistent with a previous report demonstrating that miR-409-3p is significantly downregulated in NSCLC compared with corresponding noncancerous tissues (Qu et al., 2018). Furthermore, qRT-PCR revealed that miR-409-3p expression in H460 cells transfected with si-SOD1-1 was significantly higher than that in untreated controls (Figure 4F), whereas miR-409-3p expression was significantly lower in H1299 cells transfected with pLVX-puro-SOD1 than that in controls (Figure 4F).

Next, we transfected miR-409-3p mimics into H460 cells and miR-409-3p inhibitors into H1299 cells. Subsequent qRT-PCR analysis showed that *SOD1* expression was significantly reduced in H460 cells, while it was significantly increased in H1299 cells (Supplementary Figure S5). Western blotting confirmed that SOD1 protein expression levels were also negatively regulated by miR-409-3p expression (Figure 4G). these result confirm that the expression of SOD1 and miR-409-3p was negatively correlated in NSCLC.

We predicted that miR-409-3p could partially bind to the *SOD1* 3′ UTR based on starBase v3.0 data nanlysis (Figure 4H). the results of luciferase reporter assays showed that luciferase activity was significantly reduced in H460 cells co-transfected with SOD1 3′-UTR-WT and miR-409-3p mimic (Figure 4I). In contrast, luciferase activity was significantly increased by treatment with miR-409-3p inhibitor (Figure 4J); however, luciferase activity did not vary significantly in H460 cells which co-transfected with SOD1 3′-UTR-MUT and miR-409-3p mimic or miR-409-3p inhibitor (Figures 4I,J).

### SOD1-Medoated Promotion of Cell Proliferation and Inhibition of Apoptosis Was Partly Modulated via miR-409-3p

To further investigate whether SOD1 promotes cell proliferation through miR-409-3p, we co-transfected miR-409-3p mimics and pLVX-puro-SOD1 into H1299 cells and compared cell proliferation and apoptosis with other transfected controls. The CCK-8 assay results revealed that transfection of the miR-409-3p mimic into H1299 cells reduced the proliferative capacity of cells, while co-transfected miR-409-3p mimics and pLVX-puro-SOD1 into H1299 cells led to a modest increase in proliferative capacity cpmpared with that in controls, This indicatied that miR-409-3p could significantly suppress the effects of SOD1 in promoting H1299 cell proliferation (Figure 4K). Flow cytometry demonstrated that overexpression of miR-409-3p promoted H1299 cell apoptosis. Furthermore, repression of apoptosis in response to SOD1 overexpression was relieved when cells were transfected with miR-409-3p mimic (Figure 4L), However, overexpression of miR-409-3p did not significantly reverse the effect of SOD1 in promoting cell cycle progression (Figure 4M).

### SETDB1 May Contribute to the Relationship Between miR-409-3p and SOD1 in NSCLC

**FIGURE 5:**
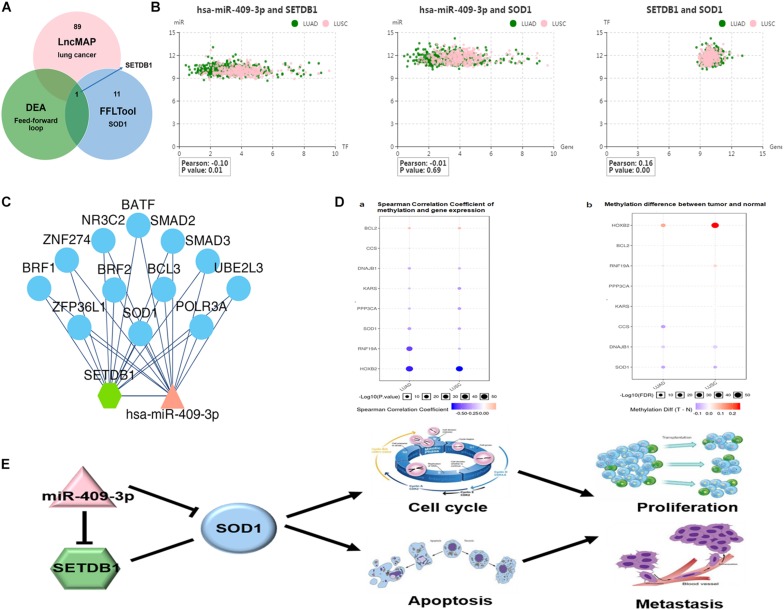
**(A)** SET domain bifurcated histone lysine methyltransferase1 (SETDB1), a transcription factor identified using the GSCALite website, influences the relationship between miR-409-3p and SOD1 in NSCLC. **(B)** Relationship among SOD1, miR-409-3p, and SETDB1. **(C)** The regulatory networks of miR-409-3p and SETDB1 both include SOD1. **((D-a)** Spearman correlation coefficient analyses of methylation and gene expression in ADC and SCC. **(D-b)** Differences in methylation between tumors and normal tissue. **(E)** Proposed feedforward loop by which SOD1 exert its functions in NSCLC cells.

Finally, since bioinformatics analyses and our experimental results show discrepancies in the relationship between SOD1 and miR-409-3p (especially the correlation between SOD1 and miR-409-3p was not significant in ADC), we used another method, GSCA Lite software (Liu et al., 2018) to analyze the association between SOD1 and miR-409-3p and determine whether other factors are involved in the regulation of SOD1 expression by miR-409-3p. As shown in Figure 5A, 90 transcription factors related to lung cancer were identified by this analysis, and 12 were highly co-expressed with SOD1, of which only SETDB1 could be regulated by miR-409-3p. Further analysis indicated that SETDB1 was significantly repressed when miR-409-3p present (Pearson = −0.1, *p* = 0.01, Figure 5B) and positively correlated with SOD1 (Pearson = 0.1, *p* = 0.16, Figure 5B). Moreover, SOD1 function was regulated by both miR-409-3p and SETDB1 (Figure 5C), while SOD1 expression was inversely proportional with the degree of methylation in both ADC and SCC (Figure 5D). Overall, we hypothesize that SETDB1 may contribute to the regulatory relationship between miR-409-3p and SOD1 in NSCLC cells via a feedforward loop (Figure 5E).

## Discussion

The SOD1 gene maps to chromosome 21q22.11 and produces a 32 kDa homopolymer antioxidant enzyme. Numerous cancers express high level of SOD1, including lung adenocarcinoma, breast cancer, and leukemia, and its elevated expression is associated with poor survival (Huang et al., 2000; Somwar et al., 2011; Papa et al., 2014). In the present study, we confirmed that expression of SOD1 is markedly elevated in NSCLC by informatics analyses. Subsequently, we used qRT-PCR, western blot, and immunohistochemistry to verify these results in both cell lines and tissues from NSCLC patients. Our results were in accordance with the bioinformatics findings as well as those of previous research, implying that SOD1 has an oncogenic role in NSCLC.

Our prognostic analysis of 196 patients with NSCLC revealed that OS and DFS were significantly worse in the group with high SOD1 expression group than those in the group with low SOD1 expression. Subgroup analysis showed that this trend remained statistically significant in both ADC and SCC, while Cox regression analysis demonstrated that SOD1 is an independent prognostic factor for OS and DFS, further supporting an oncogenic role for SOD1 in NSCLC. Nevertheless, Cox regression analysis also demonstrated that TNM stage was an independent prognostic factor for OS and DFS in patients with NSCLC, and Kaplan-Meier analysis demonstrated that SOD1 expression was significantly negatively associated with OS and DFS, however, the bioinformatics analysis indicated that SOD1 levels did not increase with TNM stage. In particular, SOD1 levels were significantly reduced in stage IV SCC, this is contrary to our experimental conclusion and bioinformatics data in ADC, because we are unable to obtain the original data of patients with stage IV SCC in the database. Thus, we speculate this part of the patients may have heterogeneity or data differences, which may lead to the negative effect on the prognosis. SOD1 probably be more meaningful to ADC. This requires further research on a large amount of samples. In addition, SOD1 was also differentially expressed in pan-cancer and normal tissues, indicating that it is tissue-specific and may play different or even opposite roles in different types of cancer. Although SOD1 shows highly expressed in many cancers and functions as an oncogene, it is still need to be furtherly studied whether SOD1 acts as the similar role in pancreatic cancer.

High SOD1 expression can promote tumor cell proliferation through multiple mechanisms. Glasauer et al. (2014) reported that SOD1 inhibition reduced antioxidant protein activity, leading to increased intracellular H_2_O_2_, which induced p38 MAPK-mediated A549 cell death, while inhibition of SOD1 in normal bronchial epithelial cells had no effect. Another group found that SOD1 inhibition increased reactive oxygen species (ROS) levels, resulting in increased DNA double strand breaks and leading to selective killing of RAD54B-deficient colorectal cancer cells (Sajesh et al., 2013). Li et al. designed a small molecule inhibitor, which selectively represses SOD1, and promotes cancer cell apoptosis via regulation of the ROS signaling network (Li et al., 2019a). Chang et al. found that up-regulation of SOD2 could increase ROS levels and decrease SOD1 expression, leading to cyclin D1 up-regulation and G1-phase cell cycle arrest. Eventually, activated caspase-3 and subsequent apoptosis in H1299 cells (Chang et al., 2018). SOD1 deficiency was shown to leads to increased oxidative stress, which can induce DNA hypomethylation in prostate tissue (Bhusari et al., 2010). Therefore, we speculate that SOD1 expression in cancer cells promotes growth and inhibits apoptosis, primarily via its antioxidantive capacity (Somwar et al., 2011; Reddi and Culotta, 2013; Glasauer et al., 2014; Wu et al., 2019). Of course, Other regulatory mechanisms are also likely to be involved; for example, SOD1 induces DNA damage and triggers the apoptotic response by activating p53 (Barbosa et al., 2010). Our results demonstrate that down-regulation of SOD1 can significantly inhibit the proliferation, invasion, and migration of NSCLC, whereas SOD1 over-expression had opposite effects. Our flow cytometry analysis verifies that alterations in SOD1 expression can promote tumor cell proliferation and metastasis by influencing the cell cycle and apoptosis, consistent with previous research results (Glasauer et al., 2014; Chang et al., 2018).

Substantial progress has been made in determining the function of SOD1 in modulating tumor cell proliferation and metastasis, however, little is known about how and which transcription factors (including miRNAs or long non-coding RNAs) could regulate *SOD1* gene transcription or translation (Bao et al., 2019). Afonso et al. (2006) identified TNF-α as a transcription factor that inhibits SOD1 transcription, translation, and promoter activity through binding to a proximal promoter sequence 157 bp upstream of the *SOD1* transcription initiation site. Moreover, AUF-1 was shown to bind to the *SOD1* 3′ UTR and primarily promotes SOD1 protein translation, leading to increased SOD1 protein expression (Zhang et al., 2015). Other mechanisms also regulate SOD1, including oxidative stress and phosphorylation. Chang et al. (2018) reported that up-regulation of SOD2 can increase ROS levels and decrease SOD1 expression in NSCLC H1299 cells. While, Tsang et al. (2018) found that the mechanistic target of rapamycin complex 1 (mTORC1) regulates SOD1 activity through reversible phosphorylation at S39 in yeast and T40 in humans in response to nutrient availability. Here, we used bioinformatics to analyze whether any NSCLC-related microRNAs could bind to the *SOD1* 3′ UTR, the result identified miR-409-3p as being associated with both NSCLC and SOD1, and able to bind to the 3′ UTR of *SOD1*, and showed that *SOD1* mRNA is a target of miR-409-3p. Based on the binding sequence, we designed a luciferase reporter assay and the results confirmed that miR-409-3p can bind directly to the *SOD1* 3′ UTR.

There is good evidence that miR-409-3p functions as a tumor suppressor in several cancers, for example, it is frequently downregulated in colorectal cancer, and acts as a tumor inhibitor by directly targeting the 3′ UTR of *NLK* (Liu et al., 2015). The expression of miR-409-3p is reduced in papillary thyroid carcinoma and may negatively regulate cell proliferation and cell cycle progression through repression of its target gene, cyclin D2, in these tumors (Zhao et al., 2018). Further, miR-409-3p expression is also decreased in tongue squamous cell carcinoma, and suppresses the proliferation, invasion, and migration of the tongue squamous cell carcinoma cells by targeting *RDX* (Chen and Dai, 2018). In the present study, we demonstrated that miR-409-3p is significantly downregulated in NSCLC cells and tissues, consistent with previous reports (Wan et al., 2014), In addition, we showed that miR-409-3p can negatively regulate SOD1 mRNA and protein levels in NSCLC cells. To further explore whether miR-409-3p can negatively regulate SOD1 functions in NSCLC cells, we designed a rescue experiment. We cotransfected miR-409-3p mimic and pLVX-puro-SOD1 into H1299 cells, and showed that overexpression of miR-409-3p could reverse the effects of SOD1 in promoting cell proliferation and repressing apoptosis. This consequence implied miR-409-3p can inhibit SOD1 activity and expressions in NSCLC. However, miR-409-3p did not inhibit the effect of SOD1 on cell cycle progression. We speculate that the effect of SOD1 on NSCLC cell cycle progression was not significantly affected by miR-409-3p, or that other regulatory factors may be involved in this regulatory process. However, this needs further experimental confirmation.

Bioinformatics analysis showed that miR-409-3p and *SOD1* expression levels were negatively correlated; but, the degree of correlation was modest, and was not significant in ADC. We believe that there may be other regulatory factors (such as transcription factors or lncRNAs) (Zhou et al., 2016) that coordinately regulate SOD1 expression with miR-409-3p. For example, increasing evidence has highlighted lncRNAs may play a critical role in tumorigenesis and prognosis of NSCLC (Sun et al., 2014; Zhou et al., 2015), and we also found that lncRNA-CBR3-AS1 exert oncogenic functions in NSCLC by targeting SOD1 (data not yet published) and this will be discussed in other articles. In this study, we assume a transcription factor could be affected by miR-409-3p or SOD1 via epigenetic mechanism, constituting a feedforward loop to regulate SOD1 function in NSCLC. Based on this hypothesis, we used GSCALite software to identify 90 lung cancer-related transcription factors related, among which only SETDB1 was associated with both SOD1 and miR-409-3p. We also found that expression of SETDB1 and SOD1 were highly correlated, and that SETDB1 levels were significantly negatively correlated with miR-409-3p. In addition, we found SOD1 expression can repress methylation in NSCLC, this consistent with findings that SOD1 knockdown induces oxidative stress and DNA methylation loss in prostate cancer (Bhusari et al., 2010), The underlying mechanism is likely that inhibition of reactive oxygen species (ROS) leads to an increase in SETDB1, as Park et al. (2019) reported that piperlongumine (Huang et al.) can downregulate SETDB1 to selectively kill breast cancer cells via accumulation of ROS, while the ROS inhibitor N-acetyl cysteine could recover the decrease in SETDB1 expression induced by PL. Consequently, we believe that SOD1 may maintain SETDB1 overexpression by down-regulating ROS levels. Our bioinformatics analyses also indicated that both SETDB1 and miR-409-3p are both related to SOD1 function, and SOD1 may associate hypomethylation of NSCLC. This suggests that miR-409-3p, SETDB1, and SOD1 form a feedforward loop to regulate cell cycle progression and apoptosis in NSCLC.

SETDB1 is an H3K9-specific histone methyltransferase that has been described as a repressive transcription factor involved in methylation and silencing of various genes (Fei et al., 2015; Riviere et al., 2016; Karanth et al., 2017). SETDB1 levels are greatly increased in numerous cancers, including lung cancer (Lafuente-Sanchis et al., 2016; Chen B. et al., 2018), and is an important epigenetic regulator involved in control of histone methylation in tumorigenesis, dysregulation of histone methylation, and aberrant miRNA profiling, contributing to tumorigenesis and progression (Chen Y. et al., 2018; Michalak et al., 2019). Moreover, DNA methylation can directly influence miRNA biogenesis (Glaich et al., 2019). Numerous microRNAs can be epigenetically silenced by DNA methylation of their promotor regions. For example, down-regulation of miR-145-5p can be attributed to methylation of the miR-145 promoter in brain metastasis of lung cancer (Donzelli et al., 2015), while Wang et al. found that H3K27me3 can bind to the miR-145 core promoter region to co-regulate *LASP1* expression in colorectal cancer cells (Wang et al., 2016). Histone methylation also mediates decreased expression of miR-449a via SUZ12 in NSCLC (You et al., 2015). Methylation of the promoter region inhibits the expression of miR-520c-3p, which functions as a novel tumor suppressor in lung adenocarcinoma (Li et al., 2019b). Furthermore, SETDB1 expression can also be regulated by microRNAs. Ectopic expression of miR-29 family molecules significantly decreases SETDB1 expression at both mRNA and protein levels by binding to the *SETDB1* 3′ UTR in NSCLC cells (Wong et al., 2016). Moreover, SETDB1 knockdown may suppress breast cancer progression, at least partly through miR-381-3p-related regulation, as SETDB1 is a verified target of miR-381-3p (Wu et al., 2018). In the present study, we speculate that SETDB1 may either promote the methylation of the miR-409-3p promoter region, or that miR-409-3p may bind to the *SETDB1* 3′ UTR, resulting in mutual regulation; however, this requires further experimental verification. Our research still has limitations, the interaction between SETDB1 and miR-409-3p, SETDB1 and SOD1 needs further study.

In summary, we have shown that SOD1 is overexpressed in NSCLC cell lines and tissues and that elevated SOD1 expression can stimulate cell proliferation and metastasis, likely through promotion of cell cycle progression and suppression of apoptosis. Furthermore, we also shown that miR-409-3p can bind to the *SOD1* 3′ UTR to repressing SOD1 expression and reversing its oncogenic effects. Meanwhile, our results suggest that SETDB1 may contribute to the regulation of miR-409-3p and SOD1 expression and functions in NSCLC cells, and SOD1 functions is likely exert via a miR-409-3p/ SETDB1/SOD1 feedforward loop. Hence, our findings provide a new insight into regulatory networks in NSCLC.

## Data Availability Statement

The datasets used and analyzed in this study are all included in the article.

## Ethics Statement

This study was approved by the Ethics Committee of The Third Affiliated Hospital of Harbin Medical University, Harbin, China. All patients gave their written consent before inclusion in the study.

## Author Contributions

SL and XX carried out the study and wrote the manuscript. SL, BL, JX, and SH helped most of the experiments. NZ, HW, and CG contributed to verify the results and revise the manuscript. HW and JL contributed to statistical analysis. All authors read the manuscript and approved the final version.

## Conflict of Interest

The authors declare that the research was conducted in the absence of any commercial or financial relationships that could be construed as a potential conflict of interest.

## Supplementary Material

FIGURE S1 SOD1 expression in normal tissues. SOD1 is expressed varied in different tissues and is Tissue-specific.

FIGURE S2 Representative images of SOD1 immunohistochemical staining in NSCLC tissues. **(a)** Low expression in SCC histotype. **(b)** High expression in SCC histotype. **(c)** Low expression in ADC histotype. **(d)** High expression in ADC histotype. (×400 original magnification).

FIGURE S3 Western blot analysis of SOD1 expression in NSCLC cell lines and a normal bronchial epithelial cell line. The histogramillustrates of overexpression of SOD1 protein in NSCLC tumor tissues relative to normal lung tissues. ^∗^*P* < 0.05, ^∗∗^*P* < 0.01.

FIGURE S4 The histogramillustrates of expression of SOD1 protein levels were analyzed following transfection of SOD1 siRNA into H460 cells. ^∗^*P* < 0.05.

FIGURE S5 The histogramillustrates of relative SOD1 mRNA expression in NSCLC cells transfected with miR-409-3p inhibitor or miR-409-3p mimic. ^∗^*P* < 0.05.
