# Supplementary figures and images for: SOD1 Promotes Cell Proliferation and Metastasis in Non-small Cell Lung Cancer via an miR-409-3p/SOD1/SETDB1 Epigenetic Regulatory Feedforward Loop
Source: Front Cell Dev Biol. 2020 Apr 23;8:213. doi: 10.3389/fcell.2020.00213 (PMC7190798; doi:10.3389/fcell.2020.00213)

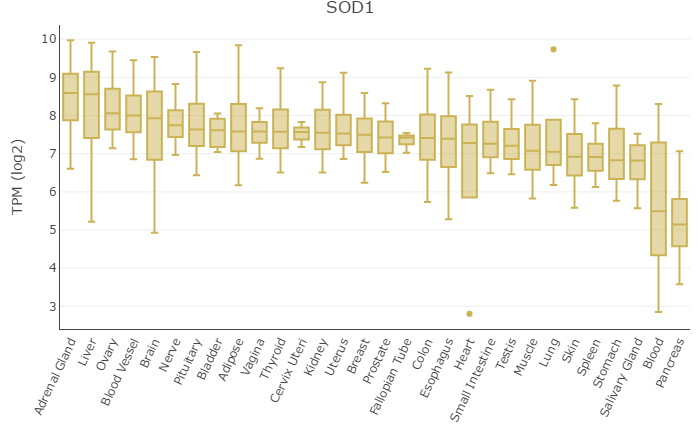

Supplement: FIGURE S1 — SOD1 expression in normal tissues. SOD1 is expressed varied in different tissues and is Tissue-specific. [file Image_1.TIF]

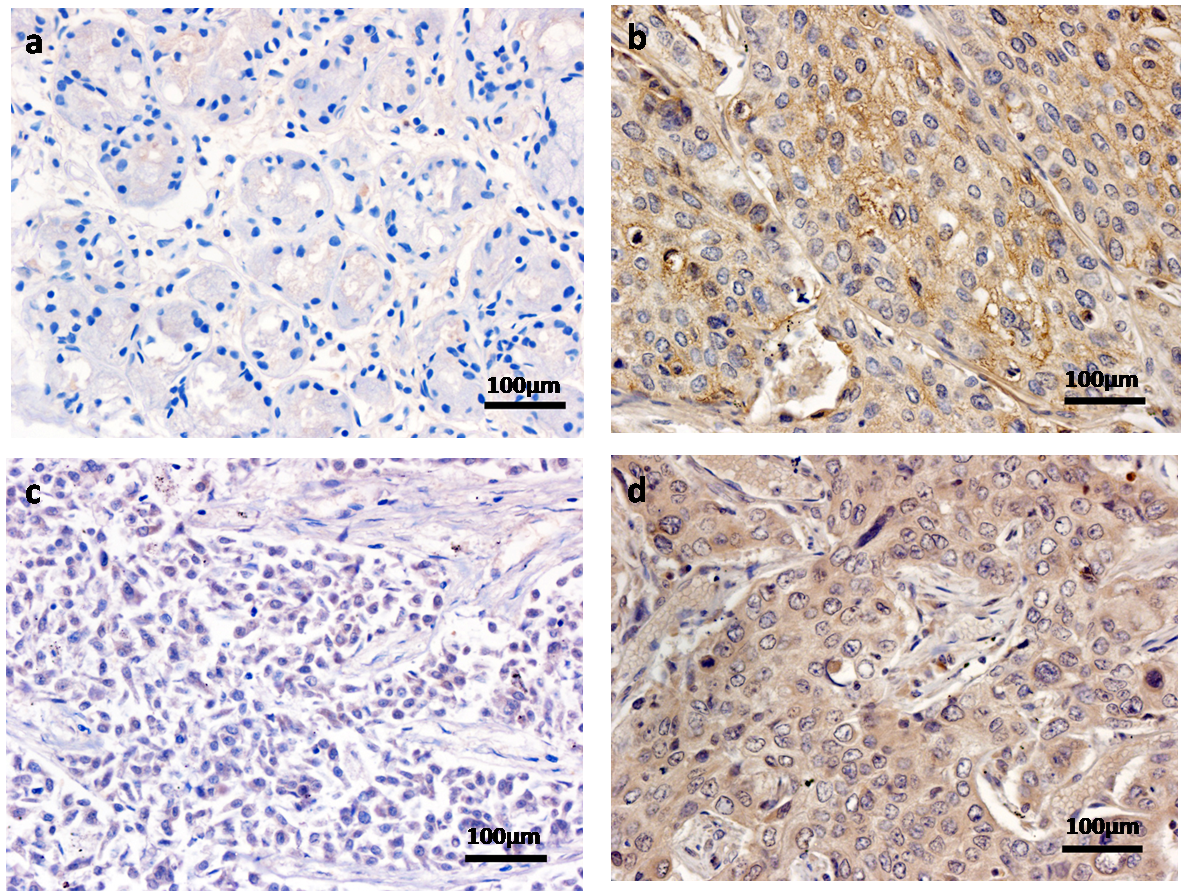

Supplement: FIGURE S2 — Representative images of SOD1 immunohistochemical staining in NSCLC tissues. (a) Low expression in SCC histotype. (b) High expression in SCC histotype. (c) Low expression in ADC histotype. (d) High expression in ADC histotype. (×400 original magnification). [file Image_2.TIF]

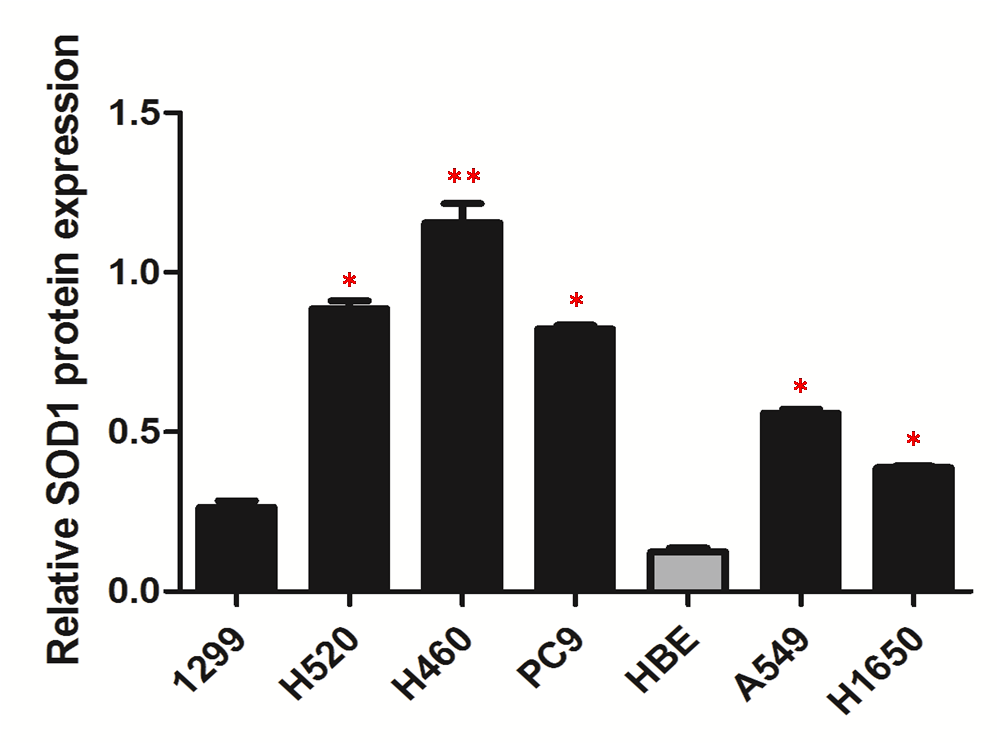

Supplement: FIGURE S3 — Western blot analysis of SOD1 expression in NSCLC cell lines and a normal bronchial epithelial cell line. The histogramillustrates of overexpression of SOD1 protein in NSCLC tumor tissues relative to normal lung tissues. ∗P < 0.05, ∗∗P < 0.01. [file Image_3.TIF]

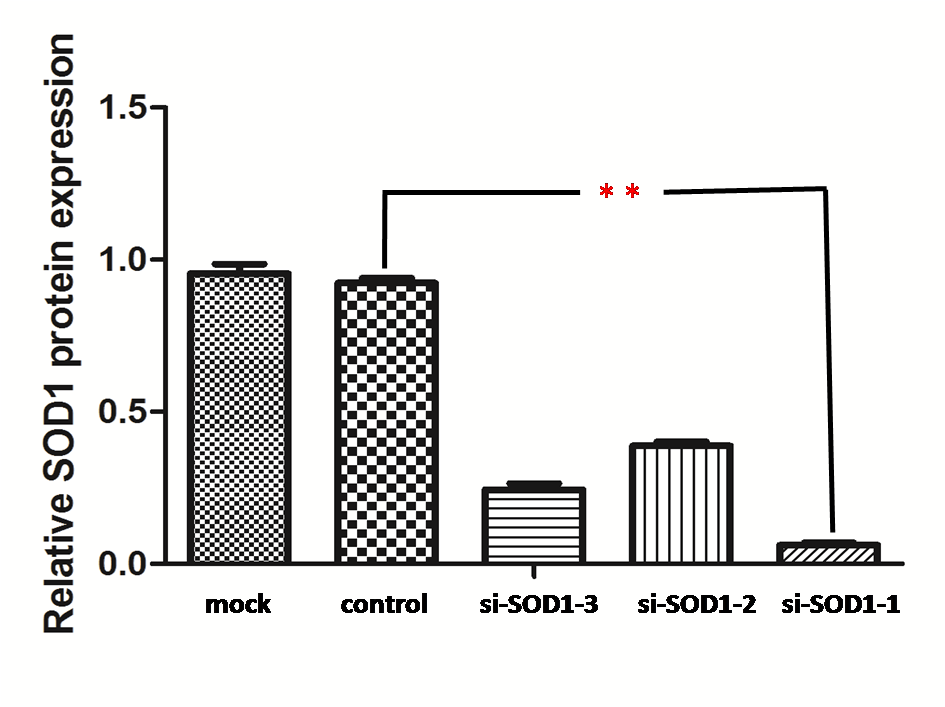

Supplement: FIGURE S4 — The histogramillustrates of expression of SOD1 protein levels were analyzed following transfection of SOD1 siRNA into H460 cells. ∗P < 0.05. [file Image_4.TIF]

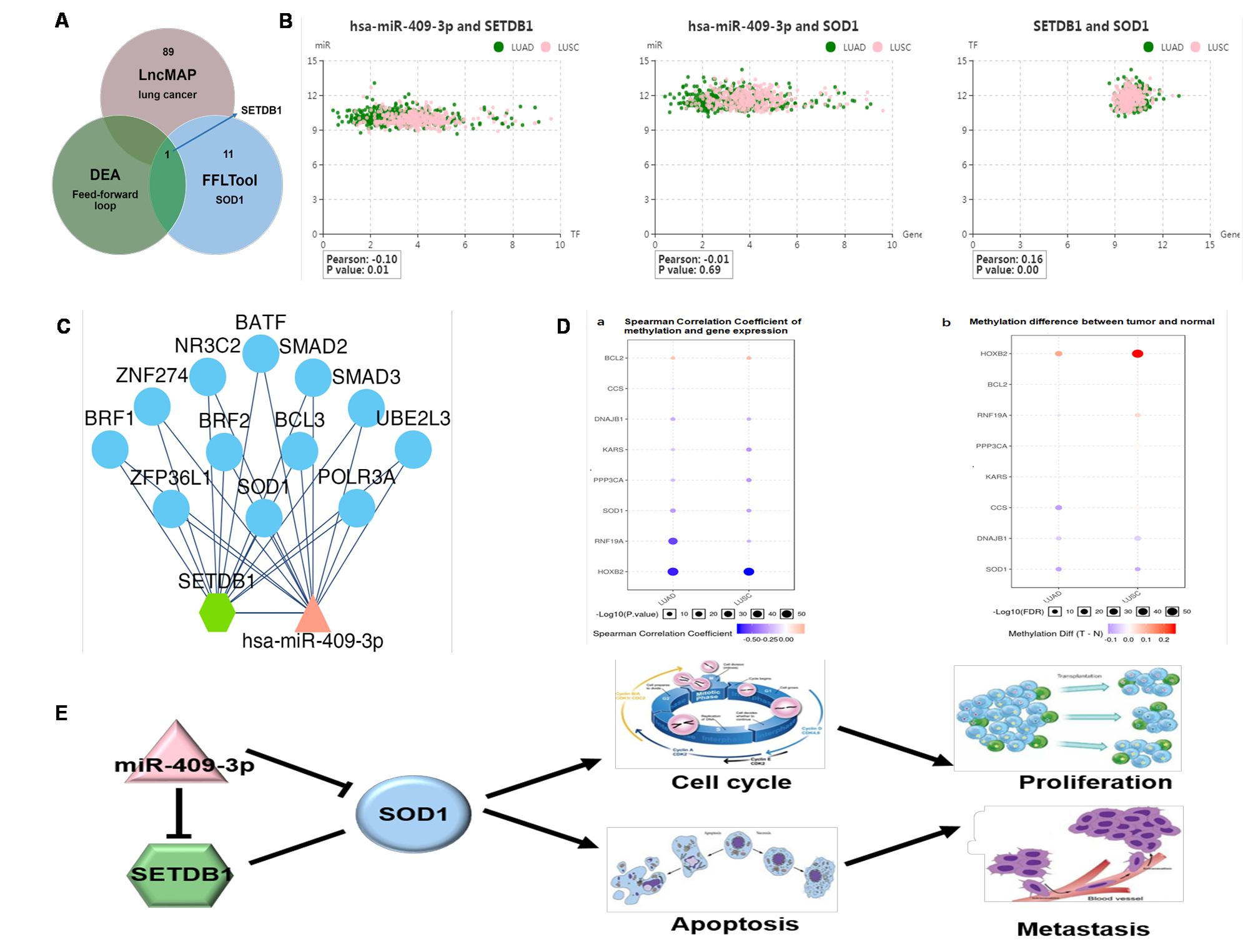

Supplement: FIGURE S5 — The histogramillustrates of relative SOD1 mRNA expression in NSCLC cells transfected with miR-409-3p inhibitor or miR-409-3p mimic. ∗P < 0.05. [file Image_5.TIF]
